# Adherence to Therapy in Patients with Multiple Sclerosis—Review

**DOI:** 10.3390/ijerph19042203

**Published:** 2022-02-15

**Authors:** Aleksandra Kołtuniuk, Justyna Chojdak-Łukasiewicz

**Affiliations:** 1 Division of Internal Medicine Nursing, Faculty of Health Sciences, Wroclaw Medical University, Bartla 5, 51-618 Wroclaw, Poland; aleksandra.koltuniuk@umw.edu.pl; 2 Department of Neurology, Wroclaw Medical University, Borowska 213, 50-556 Wroclaw, Poland

**Keywords:** adherence, medication, multiple sclerosis, disease-modifying therapy, non-adherence

## Abstract

Multiple sclerosis (MS) is a chronic, autoimmune, demyelinating disease of the central nervous system (CNS). MS is an incurable disease. The goal of disease-modifying therapies (DMT) is to slow the progression of the disease, prevent relapses and increase the patient’s overall quality of life. According to the World Health Organisation definition, adherence means the extent to which a person’s medication-taking behaviour corresponds with the agreed upon treatment recommendations from a healthcare provider. Accurate adherence is necessary for efficient treatment. Non-adherence is related to unsuccessful treatments, the risk of relapses and increased healthcare costs. The aim of this study is to present the main factors relating to non-adherence in MS patients.

## 1. Introduction

Multiple sclerosis (MS) is a chronic, autoimmune, inflammatory disease of the central nervous system. That MS affects about 2.8 million people worldwide [1]. Multiple sclerosis is an incurable condition. The most common form of the disease is relapsing-remitting MS (RRMS), which accounts for about 80% of cases, which typically have relapses of the disease. In 5–15% of cases, it is a primary progressive MS (PPMS), which is characterised by a slow progression of disability with no relapses. After 10–15 years of RRMS, the disease evolves into a secondary progressive form of MS that has a gradual disease progression [2]. The treatment of MS is based on an interdisciplinary approach. Therapy can be divided into three main groups: abortive therapies, preventive therapies and the use of symptomatic drugs. In acute exacerbations, intravenous methylprednisolone in doses of 1000 mg for three to five days is recommended as an abortive therapy. Symptomatic therapies eliminate or reduce the symptoms such as fatigue, cognitive impairment, depression and sphincter dysfunction, which impair the quality the life in patients with MS. Preventive treatment uses disease-modifying drugs (DMDs), whose goal is to decelerate the progression of the disease [3,4]. In recent years, there has been the rapid development of new therapeutic drugs. The outcome goal of treatment for RRMS is “no evidence of disease active (NEDA)” [5]. Based on this definition, “NEDA-3” means three components: no evidence of relapses, progression of disability (measured using the EDSS scale) and radiological activity in the MR images (the appearance of any new/enlarged T2W lesions and gadolinium enhancement) [6]. The NEDA concept defines the absence of disease activity. The medications that are used in long-term therapy vary in terms of their mechanism of action, route of administration, efficacy and side effect profiles. The approved DMDs for treating MS are injectable therapy, which involves interferons and glatiramer acetate; oral medications such as teriflunomide, dimethyl fumarate, fingolimod and cladribine and infused therapy, which involves natalizumab, ocrelizumab and alemtuzumab [2].

The expectations and needs of patients with MS focus on improving their quality of life and slowing the progression of the disease. Good cooperation between the patient and medical professionals is also very important. The key to effective therapy is good adherence and compliance by the patient. Non-adherence to DMDs is associated with higher rates of relapse and risk of progression of the disease [7].

The aim of this study is to present the current state of knowledge of and the factors that affect adherence in patients with multiple sclerosis. A better understanding of the causes of non-adherence could improve clinical outcomes.

## 2. Materials and Methods

This article is a review of the literature about adherence to therapy in patients with multiple sclerosis. PubMed was searched for articles from 1 January 2008 to 30 October 2021 using MEDLINE and Google Scholar. The keywords ‘adherence’ (all fields) and ‘compliance’ (all fields), ‘multiple sclerosis’ (all fields), and ‘disease-modifying treatment’ (all fields) were used to search the clinical trials and review articles and to conduct a systematic review. Additional articles were included, based on their citation in the obtained papers. A total of 190 articles were included, of which 38 were reviews, four were systematic reviews, eight were clinical trials and the remainder were other types. The final reference list was created based on their relevance to the theme of this review.

## 3. Adherence

### 3.1. Definition

Currently, in the literature, the terms ‘compliance’, ‘adherence’ and ‘persistence’ are used to define different aspects of taking medicines appropriately. Based on the WHO definition, adherence is “the extent to which patients follow medical instructions” [8]. Adherence to therapy has a primary role in achieving the benefits. Patients accepted the persistent recommendations and took the drugs, even those who were reluctant to undergo therapy. Adherence consists of three components: initiation, implementation and discontinuation [9]. The first step is initiation when the patient uses the first dose of the prescribed drug. Implementation is the time during which the patient takes the dose according to the prescribed regimen. Discontinuation is defined as the moment at which therapy stops and no more doses are taken. The term ‘persistence’ means the length of time from the initiation to the discontinuation of therapy [10]. Compliance means “the extent to which the patient adheres to the dose and takes the drug according to the prescribed schedule”. A patient’s behaviour such as taking medication, changing their diet and leading an appropriate lifestyle all have an influence on good compliance. A patient’s adherence can vary from 0, which means that the patient does not comply with the recommendations, to 100%, which means that the patient complies with the recommendations [11,12]. Optimal adherence, even as such, 100%, has been observed in clinical trials, which causes the drugs to have a better efficacy than in actual medical practice. Generally, adherence in the range 40–75% has a negative effect but this depends on the disease. In 2009, the WHO found that only 50% of patients with the chronic disease were adherent to their medications [8]. A 50% level of adherence means that every second patient is not being treated properly. The treatment of chronic disease is quite a challenge and depends on various elements. A high level of compliance and adherence to therapeutic recommendations in chronic disease is connected with a better quality of life and lower rates of hospital visits.

### 3.2. Adherence in MS

Adherence to the DMDs in MS varies widely between 41% and 93% [13,14]. For example, the adherence rate among patients who take insulin for diabetes mellitus type 2 varies between 62% and 64%, while, in group of patients who take oral medications for heart failure, it varies between 68% and 79% [10,15]. Patients with MS have a higher level of adherence during the first year of treatment with DMT than other chronic disorders such as patients with epilepsy, rheumatoid arthritis or Parkinson’s disease [16]. Patients with good adherence to DMDs have a decreased risk of relapse, a lower frequency of hospital visits and an increased quality of life compared to non-adherent patients [17,18,19,20]. In the study by Rio et al., after the cessation of therapy, patients with MS had a higher risk of relapse and progression [21]. Patients who were treated regularly with DMT had a significantly lower rate of severe relapse and lower total costs of treatment over two years [22]. The adherence to DMS therapy in MS patients is a complex problem and depends on the individual factors of each patient, the type of drug, the method of application and the co-morbidities. The available evidence indicates that many factors play a role in adherence. These factors can be divided into four groups: patient-specific factors (sex, age, cognitive status, socioeconomic status and coexisting mood disturbances); therapy-specific factors; management; and factors that are connected with healthcare systems [23].

### 3.3. Factors Affecting Adherence in MS

#### 3.3.1. Patient Characteristics

The correlation between gender and adherence is inconclusive. Most studies have shown that males are associated with a better adherence to treatment than females [24,25,26]. Generally, most studies have shown that being older is a predictor of medication-taking behaviour [7,25,27,28,29,30]. In their study, Erbay et al. demonstrated that married patients with children have a statistically lower treatment adherence [31]. When the level of education is compared, patients with a higher level of education were non-adherent during treatment more often [17,32]. Characteristics such as being in a low-income group and having high patient out-of-pocket DMT costs are also associated with poor adherence to DMTs [33]. Individual psychological factors, such as cognitive function, life satisfaction and personality, also have an impact on adherence. Forgetfulness is the most common cause of poor adherence in patients with MS. This problem may be secondary to the complexity of the treatment regimen or a cognitive impairment. Based on previous studies, forgetting to take the drug is the most or second most common reason for non-adherence [31,34,35]. Approximately 60% of patients with MS present neuropsychiatric symptoms, especially depressive symptoms [36]. Patients with depression have a significantly lower adherence rate than patients without depression [17,26,32]. McKay et al. and Tremlett et al. found that the frequent consumption of alcohol was a cause of forgetting to take the drug [35,37]. McKay et al. also showed that patients who consume a lot of alcohol had a lower level of adherence [35].

#### 3.3.2. Disease-Related Factors

The duration of the disease has a negative influence on adherence. Patients suffering from a longer course of the disease (>5 years) have a poor outcome [17,35]. Burkhard et al. demonstrated that the severity of disease also has an impact on adherence [7]. Patients with a less severe disease have a better adherence to DMT. Comorbidities are very common in patients with MS, and their frequency increases with age [38,39]. Based on the results of a systematic study, the most common concomitant problems are psychiatric disturbances (especially depression and anxiety), hypertension, lipid disturbances and chronic pulmonary disease [40]. The influence of comorbidities on treatment in patients with MS is ambiguous. These additional diseases have an impact on the clinical features, diagnosis, treatment choice (a contraindication to some drugs) and adherence [41]. Sometimes comorbidities, such as heart disease or anxiety, have prolonged the initiation of the use of DMT in patient with MS [42]. Laroni et al. showed that additional diseased were associated with increased rates of switching therapies due to an intolerance to the drugs [43].

#### 3.3.3. Drug-Related Factors

The DMTs that are used to treat multiple sclerosis are divided into oral and injectable forms of drugs. Self-injectable medications (interferons, glatiramer acetate) reduce the relapse rate by about 29–34% compared to a placebo. The treatment is related to frequent injections and adverse events, such as flu-like symptoms and skin reactions [44]. Oral therapies include fingolimod, dimethyl fumarate and teriflunomide and are related with a reduction in relapse from 46% to 58% [2]. The most common side effects for fingolimod are bradycardia or heart block, especially during the first administration. Therapy with dimethyl fumarate is related to skin flushing and gastrointestinal disturbances. New directions in the treatment of MS are monoclonal antibodies (natalizumab, ocrelizumab and alemtuzumab), which are characterised by higher efficacy than the injectable and oral drugs, as well as a 68% reduction of a relapse [45,46,47]. Adherence is highly variable and is dependent on the kind of drugs being administered. The literature has reported an adherence rate for the injectable form of DMTs, ranging from 41 to88 % in patients with MS [13]. In the study by Halpern et al., patients who were being treated with intramuscular IFNβ-1a had significantly higher odds of adherence than did patients who were on GA, SC-IFNβ-1a or IFNβ-1b [48]. Approximately 60–76% of MS patients who were treated with injection therapy (interferon beta and glatiramer acetate) adhered to the therapy for two to five years. In the study by Tremlett et al., it was found that, in a group of 97 patients who were taking an injectable form of DMT, 73% missed at least one dose, and 10% missed more than ten doses in a six-month period [37]. In another study, the same authors retrospectively assessed the medical records of 844 patients who had been treated with interferon-beta and found that 9% of patients stopped taking the drug during the first six months [37]. When a patient stopped taking the drugs due to adverse events of the therapy, this happened soon after the onset of treatment [49]. The main reasons for stopping injectable therapy were a “needle phobia”, side effects during injections (skin reactions and pain) and difficulties with self-injections [20,49]. Arroyo et al. [50] concluded that injection-related reactions were the second most common cause of non-adherence, which was confirmed by Devonshire [17]. Paolicelli reported that injection site reactions were the third most common reason for missing a dose of the drug [30]. Injection-related side effects included: skin reactions, injection anxiety and problems with injections, as well as pain after an injection. Although oral therapy seems to be a method that leads to a better adherence, it does not completely solve the problem. Patients who had been treated with fingolimod were more adherent and stopped the therapy less frequently [51,52]. Duquette et al. indicated a higher adherence at the 6-, 12- and 24-month time points in a group of patients who had been treated with oral DMF rather than self-injectable agents [53]. The same results were shown by Lahdenpera et al., as patients who used an oral DMD had a higher adherence and persistence than those that used injectable drugs [54]. According to the meta-analysis of Nicholas, 20% of patients did not adhere to a once- or twice-daily dose of oral medications, and one in four patients discontinued treatment before one year [55]. The adverse effects that are observed with oral therapies include diarrhoea, gastrointestinal discomfort and flushing. Sometimes, the side effects can be severe and can lead to discontinuing the therapy. Munsell et al. found that the method of administering the drug (self-injectable vs oral) was not a predictor of adherence [26].

#### 3.3.4. Health-Care Factors

Difficulty in obtaining information can lead to problems with adherence. Patients who are well-informed about their condition and treatment demonstrate better adherence [56]. The relationship between a patient and their physician plays a crucial role in the treatment process [57]. Treatment satisfaction can be maintained through an open and trusting relationship between the patient and their healthcare team [58]. Shared decision making between patients and clinicians promotes adherence to the treatment plan in patients with MS [59]. Communicating the risks and benefits of the prescribed DMDs and asking the patient about their values and preferences in order to determine their treatment options are the main factors that can improve a patient’s adherence to treatment. People who contacted their doctors more often (by phone, e-mail and on the day of the consultation) were significantly more adherent to the treatment [57]. In addition, nursing interventions such as telephone counselling and motivational techniques, as well as training, and support can improve the adherence to taking the drugs [60]. The study conducted by Fensterheim et al. [61] showed that patients who were new to DMT who had a one-to-one pharmacist video counselling conferencing session had significantly higher odds of being adherent. Patient-support programmes that are offered by DMT manufacturers also have a positive impact on adherence to DMTs in patients with MS [62]. Jongen et al. [63] also found associations between home care and adherence and between informal care and adherence. Adherent patients had received more frequent home and more frequent informal care than non-adherent patients. Moreover, access to DMT varies by the regions in the world. Access to therapy by the MS population in Latin America is low (9.5%-42.8%) [64]. In Europe, the access to the therapy depends on a country’s wealth. In 2013, about 70% of patients with MS from Western European countries (Portugal, Germany, etc.) were prescribed disease-modifying drugs (DMD), whereas, in Eastern European countries (Bulgaria, Poland, etc.), this only applied to only 13% of patients with MS [65].

#### 3.3.5. Cost-Related Factors

The direct and indirect healthcare costs of MS treatment are substantial [66]. The costs have risen rapidly over the last several decades and are driven by the escalating cost of DMTs [67]. High and rising costs for MS DMTs are a major concern for patients and society. The treatment costs are $90,000 a year on average [66]. The high costs of DMT place a financial burden on a healthcare system and negatively impact patients because of unaffordable out-of-pocket costs and excessive restrictions by insurance companies. Based on the results of online surveys, 40% of patients living with MS have altered the use of their DMT because of the cost. The high cost of DMTs and the process that is required to obtain insurance approvals contribute to increased symptoms and emotional distress for patients living with MS There is a strong and consistent association between patient cost-sharing levels and reduced DMT use among individuals with MS. There is also a strong association between the patient cost-sharing levels and reduced drug use and a lower adherence among patients with MS [68].

#### 3.3.6. COVID-19-Related Factors

The ECTRIMS study based on the survey confirmed that the COVID-19 pandemic has had an impact on patients with multiple sclerosis and healthcare providers for both access to care and for clinical management [69]. Patients with MS who were undergoing DMT were extremely concerned about the possibility of being infected with SARS-CoV-2. Patients with infusion DMDs were especially afraid that their medication would increase their risk of a COVID infection. The study of Zhang et al. [70], which was conducted in New York, showed that patients were generally compliant with DMT during the pandemic (only about 12.5% patients stopped undergoing DMT, and 40.8% of the respondents stated that they delayed the control appointments or laboratory tests). The study of Chertcoff et al. [71], which was conducted among patients with MS in Latin America, showed changes in the use of disease-modifying therapies (DMTs)—about 20% of the respondents did not adhere to the treatment plan.

##### Implications for Practice

Adherence is crucial for ensuring effective therapy. It is important to maintain constant contact with a patient throughout the course of treatment even by telephone so as not to overlook the signs of, for example, fatigue with the treatment [72]. If patients can talk about their worries connected with the treatment and can count on different types of support, i.e., information and psychological, they will feel much more confident, and they will take their medicine regularly. Care providers should avoid a paternalistic approach, which alienates a patient from the decision-making process. Patients whose healthcare providers respond positively and who provide feedback about their needs, views and concerns are more likely to participate in shared decision-making [59]. However, studies have shown that doctors and nurses who care for patients with MS sometimes do not have the appropriate knowledge and skills to encourage patients to actively participate in the treatment process [73]. Therefore, medical personnel should be trained in the communicative and psychological competences in communicating with patients. Patients who are active members of their therapeutic team and who participate in the decision-making process report improved satisfaction [74]. Assessing the level of treatment satisfaction is extremely important, because patients who are more satisficed adhere to the treatment plan better [31,75].

## 4. Conclusions

Non-adherence includes an interruption in taking medications or administering them in a way that is not in accordance with the prescribed schedule. Among the reasons that impede adherence are demographic factors, cognitive impairment, depression and the adverse effects of the drugs. Although the reasons that are specific for non-adherence in patients with MS include a perceived lack of efficacy, forgetfulness, inconvenience of the regimen, adverse effects/tolerability issues and injection fatigue and/or anxiety, they can also be connected with the relationship between a patient and their healthcare team. Patients who show good effects throughout the initial therapy have a better chance of achieving long-term adherence. The most important strategy is to create an individual treatment plan for each patient and to provide a high level of cooperation between the patient and their physician.
